# Information needs of people seeking fertility services in Canada: a mixed methods analysis

**DOI:** 10.1080/21642850.2021.1879650

**Published:** 2021-02-11

**Authors:** Marie-Eve Lemoine, Siobhan Bernadette Laura O'Connell, Paul Henry Grunberg, Karolanne Gagné, Carolyn Ells, Phyllis Zelkowitz

**Affiliations:** a Lady Davis Institute for Medical Research, Montreal, Canada; b University of Montreal, School of Public Health, Montreal, Canada; c Sir Mortimer B Davis Jewish General Hospital, Montreal, Canada; d McGill University, Psychology, Montreal, Canada; e Jewish General Hospital Psychiatry Research Division, Montreal, Canada; f McGill University, Biomedical Ethics Unit, Montreal, Canada; g Jewish General Hospital Institute of Community and Family Psychiatry, Psychiatry, Montreal, Canada; h McGill University, Montreal, Canada

**Keywords:** Coping, health communication, health literacy, sexual and reproductive health, stress

## Abstract

**Background:**

Infertility is a challenging experience associated with high levels of psychological distress. Many people seeking fertility services use the internet to obtain information about their conditions and treatments.

**Objectives:**

This mixed-methods study aimed to describe the information-seeking experience of people seeking fertility services with respect to the fulfillment of their individually defined information needs and explore relationships between the fulfillment of information needs and psychological outcomes.

**Methods:**

One hundred and four participants completed a survey with close-ended and open-ended questions about their experience using an informational web-based application (app) called ‘Infotility’ and about their mental well-being before and after using the app. The questionnaires administered were the *The Mobile Application Rating Scale (uMARS), the Fertility Quality of Life questionnaire (FertiQol), the Patient Empowerment Questionnaire (PEQ) and the General Anxiety Disorder 7-item Scale (GAD-7).* Eleven participants completed in-depth qualitative interviews about their experience using the app. A thematic analysis was used to interpret qualitative results and quantitization was used to dichotomize participants into those with met information needs versus those with unmet information needs. Google Analytics was used to compare participants’ reported experience with their actual use of the app.

**Results:**

The results of this study show that there is variability in the amount of information that people seeking fertility services wish to receive. Participants whose information needs were met reported improved psychological outcomes after using the app, while those with unmet needs showed no change in their psychological outcomes.

**Conclusions:**

Our results suggest that fulfilling information needs was associated with improved psychological outcomes in people seeking fertility services. Our results also suggest that individual differences in information needs should be considered when developing health educational materials.

## Introduction

Infertility refers to the inability to achieve a pregnancy within one year of having regular unprotected sexual intercourse. Experiencing infertility and engaging in fertility treatments are stressful life events that have been linked with higher levels of depression and anxiety and diminished quality of life (Chachamovich et al., 2010; Cousineau & Domar, 2007; Greil, 1997). Therefore, coping with negative feelings is an important aspect of the infertility experience.

Research has shown that over half of people seeking fertility services use the internet to complement information received from health care practitioners or to improve their understanding of fertility issues (Haagen et al., 2003; Huang, Al-Fozan, Tan, & Tulandi, 2003; Marriott et al., 2008). The ethical principle of patient autonomy demands that patients receive the necessary information to make informed decisions about treatment alternatives and lifestyle choices (Marteau, Dormandy, & Michie, 2001). Information and involvement in decision-making are also important aspects of patient-centered care (Moore, Titler, Low, Dalton, & Sampselle, 2015). Patients who are satisfied with the information they received about treatment are more likely to select treatment options based on the available scientific evidence (Mott, Stanley, Street, Grady, & Teng, 2014). In addition to serving ideals of informed choice and patient-centered care, information-seeking has been described in the literature as a coping mechanism (Miller, 1995; Timmins, 2006). Some women undergoing fertility treatments do report that seeking information about infertility and its treatment aids in coping with infertility-related stress (Benyamini et al., 2008).

Timmins (2006) used concept analysis to clarify and refine the notion of ‘information need’. This framework builds on a distinction between problem-focused coping and emotion-focused coping. Problem-focused copers attend to external events to manage the problem, while emotion-focused copers attend to internal experience to regulate their response to the problem (Lazarus & Folkman, 1984; Timmins, 2006). In this framework, problem-focused coping includes information-seeking. Once a person has adopted an information-*seeking* behavior as a response to stressful events, an information *need* arises as the success of coping depends on the success of the information-seeking. In the end, satisfying these information needs through information-seeking contributes to stress reduction (Timmins, 2006). Timmins (2006) also emphasizes that information needs are subjective and individually defined.

According to Miller (1995), patients may be characterized as ‘monitors’ or ‘blunters’ with regard to their relationship with information about their health condition and treatments. Monitors are those who attend to detailed information while blunters are those who prefer to avoid information that might cause distress. Research has shown that blunters tend to be satisfied with the basic information provided by healthcare practitioners while monitors tend to desire more information and seek a stronger knowledge base about their condition in order to gain control over the course of treatment and/or to reduce their sense of uncertainty. Therefore, those who are ‘monitors’ as opposed to ‘blunters’ may have higher information needs.

The models proposed by Miller (1995) and Timmins (2006) both support the idea that the provision of information should be tailored to the coping mechanism of the patient, with ‘monitors’ and ‘problem-focused’ patients generally requiring more extensive information than ‘blunters’ and ‘emotion-focused’ patients.

Informational websites are thought to contribute to education, empowerment, informed decision-making, sense of control, coping abilities, and better quality of life through the management of negative feelings such as anxiety and the fostering of positive feelings such as hope (Daraz, MacDermid, Wilkins, Gibson, & Shaw, 2011). According to Statistics Canada (2018), 97.1% of Canadian internet users aged between 25 and 44 own a smart phone for personal use. Since people seeking fertility services tend to fall within this age range, mobile health (mHealth) interventions have great potential for penetration in this population. In other patient populations, mHealth has been found to reduce health disparity (Nilsen et al., 2012) and improve health outcomes, attendance to medical appointments and abstinence from unhealthy habits such as smoking (Marcolino et al., 2018). In cancer patients, mHealth interventions have been shown to improve health-related quality of life (Buneviciene, Mekary, Smith, Onnela, & Bunevicius, 2021). More specifically in the context of infertility, a recent study has shown that mHealth interventions can improve nutritional and lifestyle habits in couples undergoing IVF treatments (Oostingh et al., 2020).

On average, people seeking fertility services are more educated than people receiving other types of care (Connolly, Hoorens, & Chambers, 2010; Jain, 2006). Highly educated patients tend to demand larger quantities of information compared to less educated patients (Suhonen, Nenonen, Laukka, & Valimaki, 2005; van Veenendaal, Grinspun, & Adriaanse, 1996). Accordingly, information needs and expectations with regard to information provision may be higher in the fertility patient population than in most other patient populations. Other research has also found that age and sex (Suhonen et al., 2005) as well as ethnicity (Kahlor & Mackert, 2009; Richardson, Allen, Xiao, & Vallone, 2012) may influence information needs.

Reading level is an important factor to consider when developing patient information. The Flesch-Kincaid Grade Level Formula computes the number of words per sentence and the number of syllables per word to derive the mean number of years of education required to understand a given text (Kincaid, Fishburne, Rogers, & Chissom, 1975). The need to provide patient information material at a simplified reading level has long been recognized because treatment outcomes may be threatened if patients cannot understand the basic recommendations provided to them. It is usually recommended that health information material be designed at a readability level of grade 5–8 (Cotugna, Vickery, & Carpenter-Haefele, 2005; Deatric, Aalberg, & Cawley, 2011; Health Literacy Innovations, 2008; JGH Patient Education Network, 2008; Weiss, 2006). However, it has been suggested that low readability levels are ‘paternalistic’, make for ‘infantile’ material and do not account for patients as ‘experts in their own needs and preferences’ (Coulter, 1998). More flexible guidelines have been suggested such as developing material one to three grades lower than the mean reading level of potential users (French & Larrabee, 1999). If fulfilling informational needs is a coping mechanism, there are good reasons to ensure that all patients, including the highly literate and more scientifically curious, are provided with the information they feel they need.

### The Infotility project

**Figure 1.**
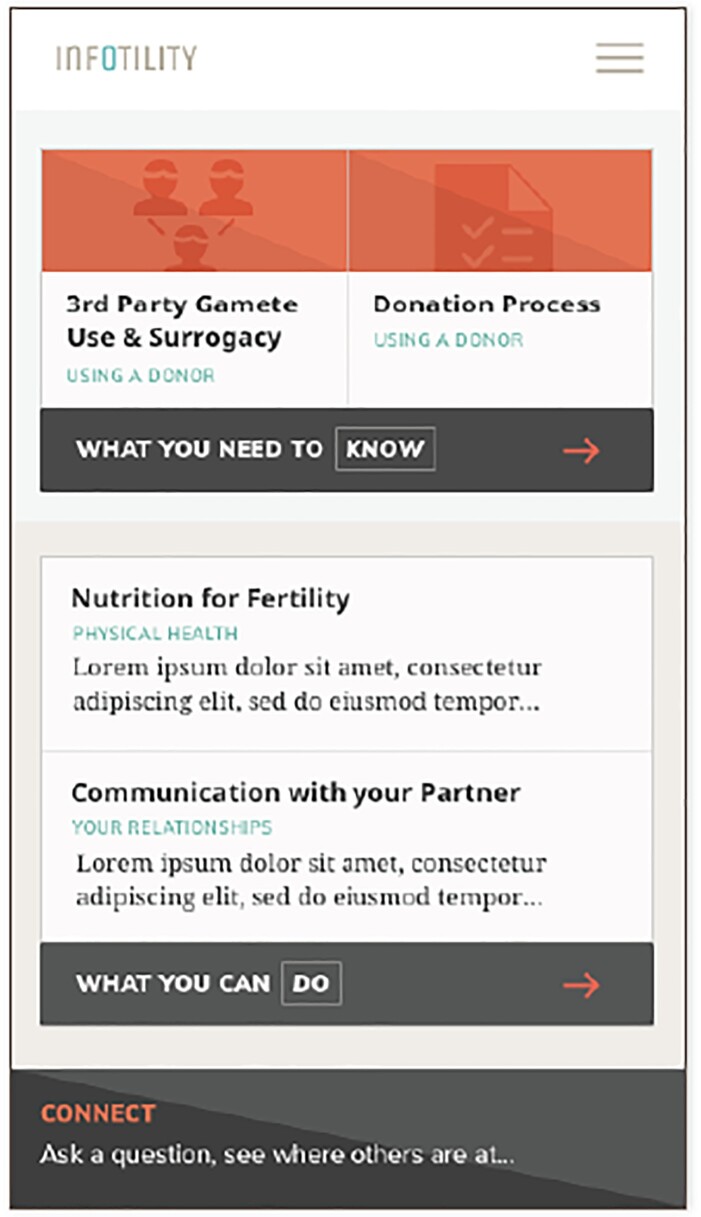
The dashboard of the *Infotility* app.

**Figure 2.**
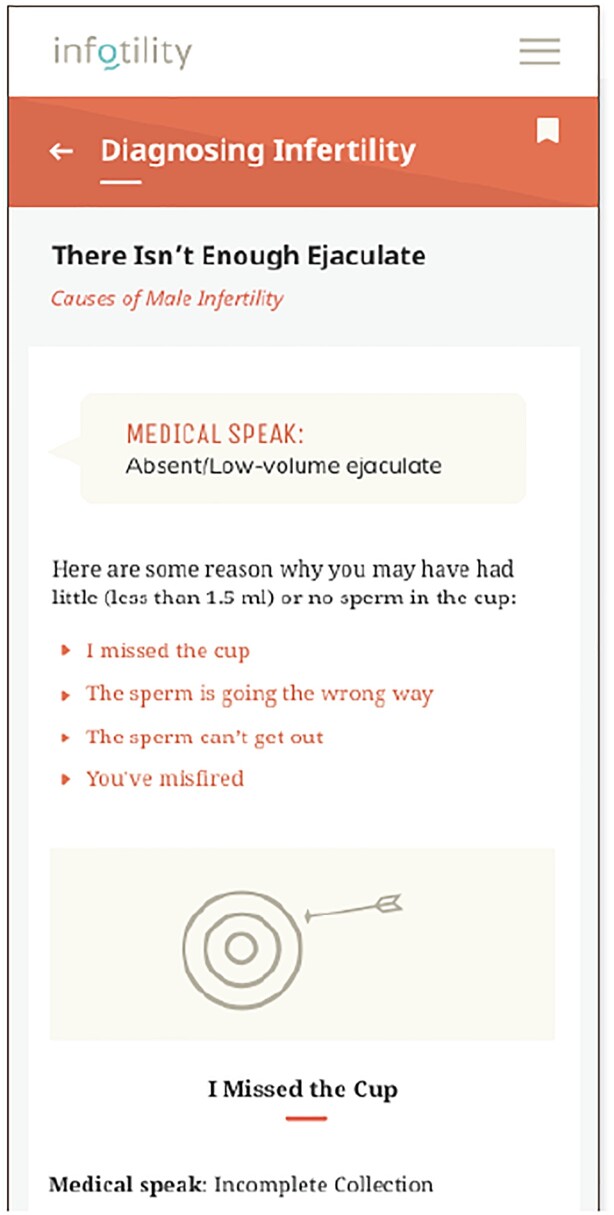
An example of an article from the *Infotility* app.

The Infotility project aimed to develop and evaluate the usability and acceptability of a mobile application (app) as a single source of reliable and understandable information for people seeking fertility services who wish to learn more about their condition and treatment. The Infotility app was created and designed by a team of clinicians and researchers from the fields of psychology, medicine, sociology, nursing, and biomedical ethics in collaboration with an app development company. The app included a home page displaying broad fertility-related topics linking to specific articles. Articles presented information in the form of text and tables, supplemented by graphics designed by the app company (see Figures 1 and 2 for an example of the app Dashboard and articles). The content on the app was informed by needs-assessment surveys of key stakeholders (people seeking fertility services and healthcare providers), as well as an extensive literature review and content analysis of existing resources. The app included 40 articles on a variety of informational topics related to reproductive health, the psychosocial challenges of infertility, fertility treatments and outcomes, and the legal and financial aspects of fertility treatment. The content was developed according to current standards for medical information tool development; words, sentences, paragraphs and sections were kept as short as possible and medical jargon was avoided as much as possible. A reading level of grade 8–10 was attained, which is slightly above common guidelines recommending grade 5–8. However, accounting for a high education level in the target population, this reading level is consistent with the above-mentioned recommendation of one to three grades lower than the mean level of the target population (French & Larrabee, 1999).

The app also included a peer-support forum where users could share their experience with fellow users and peer-supporters trained to provide emotional support. Peer-supporters were people with past experience of fertility treatment. They were trained using a peer-support manual, which was designed by the research team and reviewed by experts in the field and people seeking fertility services. They had been instructed not to provide medical information, and to encourage users to direct such questions to their physicians. All messages posted to the peer-support forum by peer-supporters were read and monitored by clinicians and researchers to ensure appropriateness and accuracy. For a more detailed description of the methods for training and evaluating the peer-supporters in this study, see Grunberg et al. (2020). Before going live with the app and recruiting participants for the current study, the app company consulted with two men and three women seeking fertility services to give feedback on the app, including its usability, design and the appropriateness of the language and content.

Within the context of the larger Infotility project, the present study aimed more specifically to describe the information needs of people seeking fertility services, to examine associations between the fulfillment of information needs, psychological outcomes and demographics, and to discuss the implications of these findings in relation to readability requirements.

## Materials and methods

### Participants

A sample of people seeking fertility services used Infotility for 8 weeks, then completed questionnaires assessing their experience with the app. Before and after using Infotility, participants were asked to answer standardized questionnaires pertaining to their psychological wellbeing. Participants were recruited at four fertility clinics in Montreal and Toronto, Canada. Recruiters approached 969 people, of whom 661 agreed to be screened for eligibility, and 505 were eligible (18 years of age or older, heterosexual, able to read English or French, have access to the Internet). The 118 people who were eligible but chose not to participate declined to do so for a number of reasons, including not interested, too busy, and mentally and/or physically distressed. Of the 387 individuals who consented to participate, 267 (69%) completed the intake questionnaires, and 220 (82%) actively used the Infotility app.^1^ The 220 participants who used the app were significantly more likely to be female than those who did not complete the intake questionnaire and those who did not actively used the app (X^2^ (1, N = 387) = 20.311, *p* < 0.000). They did not differ in any other demographic characteristic measured. Almost half (104, 47%) of the 220 app users provided qualitative feedback. A subset of participants (n = 11) were recruited to complete qualitative interviews about their experience using the app. The ethical and scientific aspects of the research were approved by the McGill University Health Center’s Research Ethics Board.

### Measures

*The Mobile Application Rating Scale (uMARS).* The uMARS is a standardized questionnaire that measures satisfaction with mobile applications through Likert-scaled questions (range 0 to 5) with an excellent internal consistency (Cronbach’s alpha = 0.90) (Stoyanov, Hides, Kavanagh, & Wilson, 2016). The Information subscale of the uMARS measures whether the participant thinks the app has high quality information from a credible source. Question 14 measures satisfaction with the quantity of information (‘Is the information within the app comprehensive but concise?’), and question 20 measures the participant’s overall star rating of the app, with a higher star rating being a better rating of the app. Three additional open-ended questions were developed by the Infotility team to be administered after completing the uMARS. These questions asked the participants to describe: (1) any topics or features that were not included on the app that they would have liked to be included; (2) what they liked best about the app; and (3) what they liked least about the app.

*Qualitative Interviews.* Qualitative interviews were conducted with the purpose of exploring participants’ experience using the app. Interviews occurred over the phone and lasted from 15 to 90 minutes. Eleven interviews were completed (3 English speaking men, 3 English speaking women, 1 French speaking man, 4 French speaking women) by two researchers, until empirical saturation was reached (Guest, Bunce, & Johnson, 2006).

*Demographics*. Background questionnaires asked participants about their sex, age, education, annual household income, ethnicity, immigration status, parity, infertility diagnosis, and number of months since first visiting a fertility specialist. Education was measured as the highest level of education completed. Income was dichotomized into below versus above the sample median household income of $80,000, ethnicity was dichotomized into white versus non-white, and parity was dichotomized into having no children or having at least one child. Infertility diagnosis was coded as male-factor infertility, female-factor infertility, mixed (both male and female factor), or undiagnosed/still in testing.

*Fertility Quality of Life questionnaire (FertiQol) – Emotional subscale.* FertiQol is a questionnaire measuring the quality of life of people experiencing fertility problems using Likert-scaled questions (range 0 to 4). Higher scores signify better fertility quality of life. The current study uses the FertiQol emotional subscale to measure the impact of negative fertility-related emotions on quality of life. The emotional subscale has a theoretical range of scores from 0 to 100 and has acceptable internal consistency (Cronbach’s alpha =0.83). The current study also uses Question 35 of the FertiQol questionnaire, which asks the participant: ‘How would you rate the quality of information you received about medication, surgery and/or medical treatment?’ (Boivin, Takefman, & Braverman, 2011).

*Patient Empowerment Questionnaire (PEQ)*. The PEQ measures patient empowerment through Likert-scaled questions (range 0 to 4). Higher scores indicate greater empowerment. The current study uses two subscales. The ‘Improved acceptance of illness’ subscale measures the participant’s level of resilience and perceived ability to cope with their illness (Cronbach’s alpha = 0.90). The ‘Confident about treatment’ subscale assesses the participant’s perceived ability to manage their illness (Cronbach’s alpha = 0.89) (van Uden-Kraan, Drossaert, Taal, Seydel, & van de Laar, 2009).

*General Anxiety Disorder 7-item Scale (GAD-7).* The GAD-7 assesses symptoms of anxiety in the past two weeks and is made up of 7 Likert-scaled questions with a total score ranging from 0 to 21. Higher scores indicate higher levels of anxiety. The GAD-7 has excellent internal consistency (Cronbach alpha = 0.92) and good convergent validity with the Beck Anxiety Inventory (*r* = 0.72) (Kroenke, Spitzer, Williams, Monahan, & Lowe, 2007).

*App engagement and Google Analytics.* Google Analytics data tracked the actual app usage of each participant. The current study focused on two indicators of engagement: (1) the total unique informational page views, and (2) the average time (seconds) spent on an informational page. We also use the following indicator as a measure of satisfaction with the information on the app: (3) whether the participant clicked ‘no’ or ‘yes’ to the question ‘did you find this page helpful?’ at the bottom of at least one informational page.

### Research design and Analytic Strategy

The Infotility project was designed to collect rich quantitative and qualitative data through self-report questionnaires, short-answer open-ended questions, and in-depth qualitative interviews. For the present study, a convergent mixed methods approach was used to explore the information needs of participants and the characteristics that may influence these needs. Convergent designs involve gathering qualitative and quantitative data independently, analyzing them separately and merging them at the interpretation stage by highlighting complementarity and discrepancies in the findings. Compared to other mixed-method designs, convergent designs are particularly relevant when both data sets are equally important to the research and they provide a more comprehensive analysis of the research problem (Creswell 2014). For this study, the qualitative question was ‘how did people seeking fertility services describe their fertility-related information needs and information-seeking experience?’ The quantitative question was ‘how were demographic and psychological factors related to the fulfillment of information needs?’ The qualitative and quantitative data were combined and compared at the interpretation stage. The complementary aspects and discrepancies identified inform a discussion about the relevance of more flexible guidelines with regard to readability standards.

Qualitative thematic analysis (Braun & Clarke, 2014) was used to analyze the transcripts from the qualitative interviews, and the qualitative section of the uMARS. Codes were used to describe the experience of patients seeking information, for the purpose of answering our first question: How did people seeking fertility services describe their fertility-related information needs and information-seeking experience? For the purposes of this paper, all French quotations have been translated into English.

Quantitization (Sandelowski, Voils, & Knafl, 2009; Tashakkori & Teddlie, 1998) was used to translate the qualitative answers from the uMARS questionnaire into two binary categories for quantitative analyses: (1) participants remaining with unmet information needs after using the app (‘the unmet needs group’), and (2) participants whose needs had been met by the app (‘the met needs group’). This quantitization process was required because this study is a secondary analysis and a direct question about information needs having been met was not included in the primary study.

Participants were understood to have unmet information needs when responses indicated dissatisfaction with the depth of the information. The first qualitative question on the uMARS asked for specific topics or features that participants would have liked to see included. Many participants used this opportunity to express their feeling that the app in general, or one specific section, was not detailed enough (too simple, too short, missing detail, not medical enough …). These participants were categorized as having ‘unmet needs’. Information needs were considered to have been met when this question was left blank, when respondents said the app was complete as it was or when they referred only to technical features.

Question 3 asked participants what they liked least about the app. Many mentioned lack of detail as what they liked least. They were included in the ‘unmet needs’ group.

Two researchers analyzed and coded the qualitative uMARS data in order to classify the participants for the quantitization, and Cohen’s Kappa was run to determine if there was agreement between the two researchers’ coding of these samples. There was a strong agreement between the two researchers, Kappa = .918, *p* < 0.05.

This new dichotomous variable was used in the quantitative analyses to explore what factors were associated with met and unmet needs. Independent samples t-tests and chi-square tests were used to examine whether those with unmet needs differed significantly from those with met needs in demographic and fertility-related characteristics, engagement and satisfaction with the app, and psychological outcomes. Logistic regressions were used to analyze whether psychological outcomes were associated with having information needs being met, controlling for variables that were significant in bivariate analyses. Repeated measures ANOVAs were run for measures of psychological outcomes with group as the between-subjects factor (unmet needs group compared to met needs group), time as the within-group factor (from intake to follow-up), and an interaction term between group and time. The current study had a relatively small sample size and was exploratory in nature, looking for general trends and not attempting to determine clinical significance or make causal conclusions. For these reasons, no corrections for multiple comparisons were undertaken; results should be interpreted with caution. Statistical analyses were conducted using IBM SPSS Statistics 19.

## Results

### Research question #1 – participants’ description of their information needs and information-seeking experience:

104 participants left qualitative answers on the uMARS and 11 participants completed qualitative interviews. Participants with qualitative data were more likely to be female in comparison to the full sample of *Infotility* users. They did not differ in any other demographic characteristic or psychological outcomes. Participants with qualitative data were primarily women (*n* = 88, 84.6%), white (*n* = 62, 59.6%), and born in Canada (*n* = 66, 64.1%). Mean age was 35.5 years (*SD* = 5.1). A majority of participants held a graduate degree (*n* = 45, 43.3%), and approximately 75% (*n* = 78) had a university degree or higher. The majority of the sample had an average annual household income of $80,000 or more (n = 62, 60.8%). The average time from first fertility clinic visit was 18.9 months. About 34% (*n* = 35) of the sample reported female-factor infertility, 24% (*n* = 25) male-factor infertility, and 32% (*n* = 33) testing stage or unexplained infertility (Table 1).

**Table 1.** Comparing participants’ demographics and fertility-related characteristics.

	Full sample		Unmet needs		Met needs				
	n	Valid % / M (SD)	n	Valid % / M (SD)	n	Valid % / M (SD)	X^2^	t	*p*
Sex Male Female	104 16 88	15.4 84.6	55 8 47	14.5 85.5	49 8 41	16.3 83.7	.063	–	1.000
Age (24–54)	104	35.5 (5.1)	55	35.9 (5.3)	49	35.1 (5.0)	–	−.759	.449
Highest level of education High school or less Cegep, vocational University degree Graduate degree	104 9 17 33 45	8.7 16.3 31.7 43.3	55 1 12 19 23	1.8 21.8 34.5 41.8	49 8 5 14 22	16.3 10.2 28.6 44.9	8.790	–	.032*
Annual household income ($) Less than 80,000 80,000 and over	102 40 62	39.2 60.8	55 21 34	38.2 61.8	47 19 28	40.4 59.6	.054	–	.841
Ethnicity White Non-white	104 62 42	59.6 40.4	55 39 16	70.9 29.1	49 23 26	46.9 53.1	6.184	–	.017*^a^
Immigration status Immigrant Born in Canada	103 37 66	35.9 64.1	54 18 36	33.3 66.7	49 19 30	38.8 61.2	.331	–	.681^a^
Parity No children 1 child or more	104 87 17	83.7 16.3	55 46 9	83.6 16.4	49 41 8	83.7 16.3	.000	–	1.000^a^
Infertility diagnoses Male-factor only Female-factor only Mixed Still testing/Unexplained/Other	104 25 35 11 33	24.0 33.7 10.6 31.7	55 11 21 8 15	20.0 38.2 14.5 27.3	49 14 14 3 18	28.6 28.6 6.1 36.7	3.973	–	.264
Months since first seeing fertility specialist	103	18.9 (18.6)	55	17.8 (19.4)	48	20.3 (17.8)	–	.685	.495
Pregnancy achieved during study Yes No	101 80 21	79.2 20.8	52 44 8	84.6 15.4	49 36 13	73.5 26.5	1.903	–	.221

*Abbreviations.* n = number of cases; M = mean; SD = standard deviation; X^2^ = Chi-square test; t = independent samples t-test; *p* = significance value (2-sided test).

Notes*.* All coefficients are significant at *p* < .05*, *p* < .01**, *p* < .001***.

^a^ Fisher’s exact test reported.

Eight themes emerged through the analysis of the qualitative data.

1. *Information-seeking as the main reason for using the app.* In the qualitative interviews, *seeking information* was cited by almost all participants as the primary reason for using Infotility.“My aim with accepting to participate with the app was can you help me get more information and […] reassure me to fill the gaps of information I may not know I have” (#17, female).Other reasons included *feeling lonely* and *needing support*. However, these participants would still discuss information in their descriptions of their motives:“I wanted to feel less alone. I wanted to get information” (#10, female).“I would say support in fact. I thought it was a good idea to provide information” (#64, male).Some participants used Infotility’s peer-support forum to acquire information from trained peer-supporters or other patients. Their account of their experience show that information and support are intertwined in their minds:“I found the peer-support interventions they were good, but they were very careful. I guess they were very restrained. You could see they were trying to be supportive without getting into too much information, they all said go talk to your doctor[…]. It was more like, oh yeah, we hear you it happens, you’re not alone. And I don’t think it did it for me, but I think it might have helped other people … ” (#17, female).
2. *Information-seeking as a coping mechanism.* Participants identified information-seeking as a coping mechanism to overcome a sense of passivity in the context of treatments or to gain a sense of control:*“*[Physicians] wanna make the patient feel like they are in control and that they know what they are doing […]. But for us it didn’t work, we didn’t feel like we had any control over the situation. So, for us going back online was what we could do on our side, it was a way to be able to find little control” (#28, male).
3. *Unmet information needs in clinical encounters.* Participants mentioned that they were not satisfied with the amount of information provided at the fertility clinic and sought to complete their knowledge with online information.“[…] we didn’t have a doctor that was spending too much time giving us information on what’s happening. I mean each time we would meet the doctor at the fertility clinic it was literally five minutes of appointment where they would tell us this, this and this and that’s it. Each time you had questions they were like ‘no problem, ask me anytime’ and then they were like ‘you can ask the nurse as well after if you still have doubts’. And it’s like yeah but I wanna know from you. I mean I’m not saying that the doctor would not reply to our questions, she always did but it was super brief” (#28, male).
4. *The consequences of unmet information needs.* Participants conveyed that unmet information needs make for uncertainty about treatments and a sense of uninformed choice:Participant: “If I knew better, I would have asked my doctor. Maybe he didn’t think it was a good idea to start sooner with […] a procedure which would have given me a better chance”Interviewer: “So I’m hearing that throughout this process, getting more information has helped you make better decisions for yourself.”Participant: “It would have helped me if I had the information at the right time” (#56, female).
5. *The importance of reliability.* Many participants reported distrusting online information. Some added that it is difficult to judge the reliability of the information provided on available websites. In that sense, one of the most appreciated features of Infotility was the fact that the content was written and approved by researchers and health professionals collaborating directly with the clinics where participants received treatments:*“*[…] the information they were filtered through doctors. It’s not like they were randomly saying stay away from coffee because it’s not good for your sperm analysis. Someone has been validating that information. […] having someone that does the work for you and is filtering all this information through doctors; it’s just validating the information” (#28, male).
6. *The infinite nature of information needs.* Participants identified a sense that their information needs could never be met as there was always something more to learn about fertility and treatment:*“*[…] I think we can never have enough. […] you still go back online to try and find more information. There is something new that is coming up each time and you’re like I never thought about that let’s go and check online about this kind of food that can have an impact or anything like that. So you never stop searching for something throughout the process, at least in our case […]” (#28, male).
7. *Patients becoming ‘experts’ of their condition and treatment.* A few participants made distinctions between the information needs of those starting their fertility journey and those with previous treatment experience. It was suggested that the amount of information provided was sufficient for neophytes but insufficient for veterans:“The info was basic: stuff I researched at the very beginning of my infertility journey. After almost 4 years, there was no new information for me. I also found the info never went into enough detail. It was basic” (#81, female).“Most of the information I need right now is on it (since we are right at the very beginning). Not sure how helpful this would be later on as we find out more information” (#91, female).
8. *Desire for very detailed information.* The most common comment that participants gave about Infotility was that the information was not detailed enough. Participants were hoping for content that went beyond the general information given by physicians and from the internet, especially medical information. Since more than half of our qualitative sample had expressed this thought, it became the basis for further analysis through our quantitization process.“It’s brief and generic  …  maybe too much. It seems to skim over the surface and then refers to the doctor for specifics  …  But when one is undergoing fertility treatments, there is a need for medical information that may or may not apply to you. […] The app didn’t go the extra mile worth paying for by keeping a too safe distance of the medical nitty gritty” (#17, female).

Participants also articulated this thought by saying that they had seen all the content in very little time, or that the information provided was not new to them:

“Held my attention for 10–20 minutes and then I felt I had seen it all. I had no inclination to revisit. Not enough in-depth content” (#79, female).

“The information was pretty general and most of it was information I had already read up on” (#60, female).

### Research question #2 – differences between participants with unmet needs and those with met needs

On average, participants rated the language and amount of detail provided in Infotility with a score of 3.7/5 (Table 2, uMARS question 14). Our quantitization revealed that 55/104 had unmet information needs after using Infotility whereas 49/104 had met needs. Statistical analyses highlight a number of key differences between those whose information needs were unmet compared to those whose needs were met.

1. *Demographic differences.* The unmet needs group was significantly more likely to be white (X^2^ (1, N = 104) = 6.184, *p* = 0.017) and have a higher education level (X^2^ (3, N = 104) = 8.790, *p* = 0.032) than the met needs group (Table 1). While both groups were highly educated (the majority of participants in both groups had a university degree or higher), significantly more of the met needs group reported high school or less as their highest level of education. When education, ethnicity, and psychological outcomes (FertiQol, two PEQ subscales, and GAD-7) were included in logistic regression models, ethnicity remained significant, but education was no longer a significant predictor of information needs. The two groups did not differ in terms of sex, income, immigration status, parity, and length of time since first visiting a fertility specialist. There were no differences between the two groups as to whether they achieved pregnancy during the study period (Table 1).
2. *Engagement and satisfaction.* When asked to rate the information on the app, those with unmet needs rated the quality (t(100) = 2.229, *p* = 0.028) and the quantity (t(101) = 2.731, *p* = 0.007) of the information significantly lower than those with met needs. In addition, those with unmet needs gave the app a significantly lower overall star rating (t(102) = 4.038, *p* = 0.000) compared to those with met needs. Finally, 32.1% of those with unmet needs reported that at least one informational page was not helpful, whereas only 8.2% of the met needs group did (X^2^ (1, N = 102) = 8.904, *p* = .003) (Table 2).

**Table 2.** Comparing participants’ engagement and satisfaction with the app.

	Full sample		Unmet needs		Met needs				
	n	Valid % / M (SD)	n	Valid % / M (SD)	n	Valid % / M (SD)	X^2^	t	*p*
Unique informational page views	102	13.3 (11.6)	53	15.8 (12.7)	49	10.5 (9.6)	–	−2.391	.019*
Avg. time on a page (seconds)	102	47.7 (36.9)	53	55.1 (36.8)	49	39.8 (35.6)	–	−2.123	.036*
‘Did you find this page helpful?’ ^b^ Clicked ‘yes’ at least once Clicked ‘no’ at least once	68 21	66.7 20.6	38 17	71.7 32.1	30 4	61.2 8.2	1.257 8.904	–	.298 .003**^a^
uMARS Information subscale	92	4.0 (0.6)	48	3.9 (0.6)	44	4.1 (0.6)	–	2.229	.028*
uMARS Q14	103	3.7 (0.9)	55	3.5 (1.0)	48	4.0 (0.8)	–	2.731	.007**
uMARS Q20 Overall star rating	104	3.5 (0.7)	55	3.2 (0.7)	49	3.7 (0.6)	–	4.038	.000***

*Abbreviations.* uMARS = Mobile application rating scale (user version); uMARS Q14 = ‘Is the information within the app comprehensive but concise?’; n = number of cases; M = mean; SD = standard deviation; X^2^ = Chi-square test; t = independent samples t-test; *p* = significance value (2-sided test).

Notes*.* All coefficients are significant at *p* < .05*, *p* < .01**, *p* < .001***.

^a^ Fisher’s exact test reported.

^b^ These are not distinct groups, as participants could click ‘No’ or ‘Yes’ to the question ‘Did you find this page helpful?’ at the bottom of each article as many or as few times as they wished.

Although the unmet needs group had lower satisfaction, they had higher levels of engagement than those with met needs. The unmet needs group visited significantly more informational pages (t(100) = −2.391, *p* = .019), and spent significantly more time on each page compared to the met needs group, t(100) = −2.123, *p* = 0.036 (Table 2).

1. *Psychological outcomes at intake.* The unmet needs group scored significantly higher at intake on the FertiQol emotional subscale and PEQ acceptance of illness and confident about treatment subscales, and significantly lower on the GAD-7 compared to the met needs group (Table 3). These results remain significant after controlling for education and ethnicity, the demographic variables significant in bivariate analyses. There was no significant difference in ratings of the quality of information about medication, surgery, and/or medical treatment before the study (Table 3, FertiQol Q35).
2. *Changes in scores from intake to follow-up questionnaires*.

**Table 3.** Comparing participants’ fertility-related quality of life, patient empowerment and anxiety at intake and follow-up.

	Full sample		Unmet needs		Met needs			
	n	Valid % / M (SD)	n	Valid % / M (SD)	n	Valid % / M (SD)	t	*p*
*Intake questionnaires*								
FertiQol Emotional subscale	102	52.4 (22.7)	54	57.1 (21.5)	48	47.0 (23.0)	−2.280	.025*
FertiQol Q35	101	2.5 (0.9)	54	2.6 (0.9)	47	2.4 (1.0)	−1.008	.316
PEQ Acceptance of illness	101	2.3 (0.9)	53	2.5 (0.8)	48	2.1 (0.9)	−2.767	.007**
PEQ Confident treatment	102	3.0 (0.6)	54	3.1 (0.7)	48	2.8 (0.6)	−2.352	.021*
GAD-7 Total score	100	7.3 (5.8)	53	6.0 (5.3)	47	8.8 (6.1)	2.475	.015*
*Follow-up questionnaires*								
FertiQol Emotional subscale	97	52.1 (21.7)	51	52.1 (19.9)	46	52.0 (23.7)	−.030	.976
FertiQol Q35	99	2.3 (1.1)	51	2.3 (1.0)	48	2.3 (1.3)	−.182	.856
PEQ Acceptance of illness	100	2.4 (0.9)	52	2.4 (0.9)	48	2.3 (0.9)	−.978	.331
PEQ Confident treatment	98	3.0 (0.5)	51	3.1 (0.5)	47	3.0 (0.6)	−.619	.538
GAD-7 Total score	96	5.9 (4.9)	53	5.3 (4.0)	43	6.5 (5.7)	1.159	.250^a^

*Abbreviations.* FertiQol = Fertility Quality of Life Questionnaire; FertiQol Q35 = ‘How would you rate the quality of information you received about medication, surgery, and/or medical treatment?’; PEQ = Patient Empowerment Questionnaire; GAD-7 = General Anxiety Disorder 7-item Scale; n = number of cases; M = mean; SD = standard deviation; t = independent samples t-test; *p* = significance value (2-sided test).

Notes*.* All coefficients are significant at *p* < .05*, *p* < .01**, *p* < .001***.

^a^ Equal variances not assumed.

**Figure 3.**
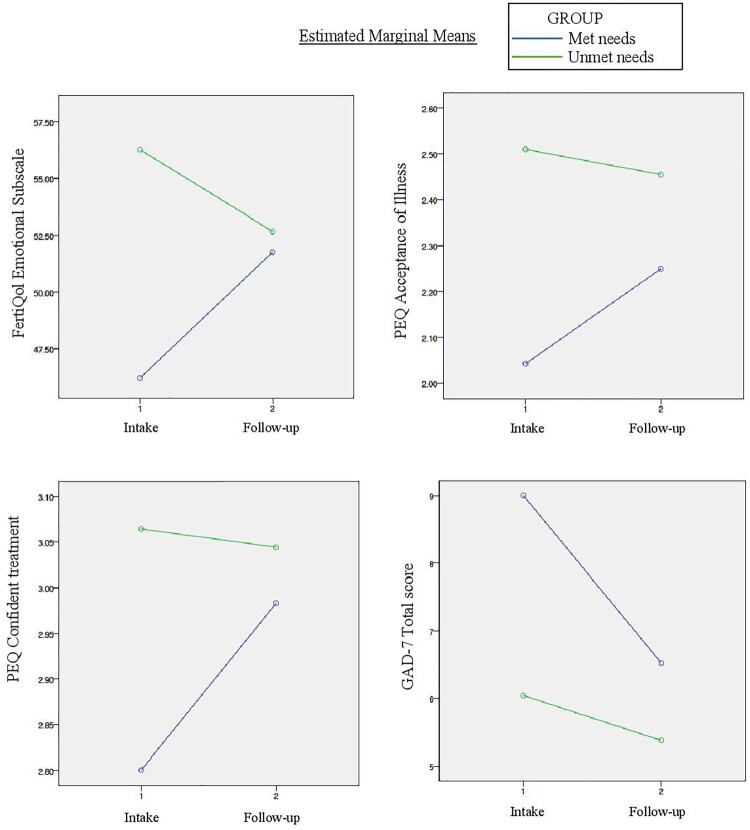
Interaction effect between group (unmet versus met needs) and time (intake to follow-up) on psychological outcomes (repeated measures ANOVAs)

After 8 weeks of using Infotility, there were no significant differences between the two groups in the FertiQol (emotional subscale), PEQ, or GAD-7 (Table 3). Results from the repeated measures ANOVA (Table 4) showed that the interaction of time (from intake to follow-up) and group (having met needs versus unmet needs) was statistically significantly associated with the FertiQol emotional subscale. The interaction of time and group was also associated with the PEQ subscales and GAD-7 scores, with *p*-values approaching significance (*p* < 0.10). Figure 3 shows the interaction effect between group and time on the FertiQol emotional subscale, PEQ acceptance of illness and confident about treatment subscales, and GAD-7. In general, those with met needs improved in psychological outcomes throughout the study period, while those with unmet needs did not change in their psychological outcomes throughout the study period.

**Table 4.** Comparing participants’ changes in mean scores from intake to follow-up (repeated measures ANOVA).

Effect	MS	df	F	*p*	Partial Eta Square
FertiQol Emotional subscale					
Time (Intake to follow-up)	46.062	1	.383	.538	.004
Time x met needs	989.044	1	8.214	.005**	.081
Error	120.410	93			
PEQ Acceptance of illness					
Time (Intake to follow-up)	.282	1	1.302	.257	.014
Time x met needs	.834	1	3.859	.052^+^	.039
Error	.216	95			
PEQ Confident treatment					
Time (Intake to follow-up)	.322	1	2.425	.123	.025
Time x met needs	.499	1	3.762	.055^+^	.038
Error	.133	95			
GAD-7 Total score					
Time (Intake to follow-up)	113.751	1	10.061	.002	.100
Time x met needs	37.708	1	3.335	.071^+^	.035
Error	11.307	91			

*Abbreviations.* FertiQol = Fertility Quality of Life Questionnaire; PEQ = Patient Empowerment Questionnaire; GAD-7 = General Anxiety Disorder 7-item Scale; MS = mean square; df = degrees of freedom; F = F-statistic from repeated measures ANOVA; *p* = significance value.

Notes*.* All coefficients are significant at *p* < .05*, *p* < .01**, *p* < .001***, *p* < .1^+^

## Discussion

Our qualitative results show that people seeking fertility services described information-seeking as a coping mechanism and our quantitative data suggest that information needs being met correlates with improved psychological outcomes. This suggests that information-seeking may be an effective coping mechanism when individually defined information needs are met. People in the unmet needs group were more likely to be white and to be more educated. Therefore, white ethnicity and a high education level may be indicators of a high need for information.

### Information-seeking as a coping mechanism

Our interview participants described their motivations for using the app in terms of information needs and support needs. This is consistent with the results of prior studies (Haagen et al., 2003; Kahlor & Mackert, 2009; Read et al., 2014). However, the information discourse was predominant in our interviews, suggesting that the information section might have been the main driver for agreeing to participate in our study. Some responses indicated that participants may not have considered information and support as separate goals. Indeed, information is mentioned as a coping mechanism, and the peer-support forum was used with the intent to find information.

Across our measures of psychological outcomes, a clear pattern emerged when comparing the met needs group with the unmet needs group. At intake, the unmet needs group was more empowered, had better fertility quality of life (emotional subscale), and was less anxious than the met needs group, however at follow-up, there were no longer any significant differences between the two groups. The psychological outcomes of the met needs group were improved during the course of the study while the psychological outcomes of the unmet needs group did not change (see Figure 3). Therefore, it is possible that having their information needs met by using the app contributed to an improved sense of empowerment with regard to fertility treatment, improved fertility quality of life (emotional subscale) and decreased anxiety. However, there is a need for experimental designs to further examine this relationship.

Our qualitative findings suggest that our participants used information-seeking as coping mechanism to gain control and to feel less passive during treatment. Miller (1995) also reported that ‘monitors’ may be motivated by a desire for increased control and for an active role in decision-making. Other studies have established a positive relationship between the acquisition of information and a sense of control (Ream & Richardson, 1996), and between a sense of control and psychological outcomes such as perceived quality of life and depressive symptoms (Abeles, 1991; Lachman & Weaver, 1998). Therefore, experimental research could hypothesize that fulfilling information needs contribute to improved psychological outcomes by improving patients’ sense of control.

In our qualitative survey, participants from the unmet needs group reported that they already knew a lot of the information that was on the app before using it. This indicates that they may have started out with a stronger knowledge base. This could explain the unmet needs group’s higher expectations for the level of detail to be provided for information on the app to be found useful. A stronger knowledge base combined with better psychological outcomes at intake for the unmet needs group would be consistent with more prevalent ‘problem solving’ coping mechanism and ‘monitor’ information-seeking behavior in this group. Experimental research with these constructs as independent variable would be required to confirm this hypothesis.

Improvement in empowerment, fertility quality of life (emotional subscale) and anxiety are in line with previous literature saying that fulfilling information needs contributes to better coping (Miller, 1995; Timmins, 2006). It is not possible to confirm that these improvements were attributable to participating in the study as improvements could be attributable to external life events. Previous research has shown that the success of fertility treatments improves infertile women’s symptoms of distress (Greil, McQuillan, Lowry, & Shreffler, 2011). However, there were no differences between the two groups in whether or not they achieved pregnancy during the study. Therefore, the improved psychological outcomes of the met needs group are not attributable to better success in fertility treatments. Satisfaction with the information provided may also have been influenced by individual characteristics, such as lower neuroticism or trait optimism, that were not measured in this study.

Previous research with cancer patients has shown that internet information-seeking can lead to increased positive feelings such as hope or a sense of being better informed, but it can also trigger negative feelings such as confusion and anxiety (Helft, Hlubocky, & Daugherty, 2003). Such feelings were not reported in our qualitative results, and our quantitative analysis did not reveal any significant deterioration of psychological outcomes. Therefore our study does not support the notion that internet information-seeking may have detrimental effects for people seeking fertility services.

### Meeting information needs

Our participants recognized that the internet in general is not the most reliable source of information. They were able to identify the credible and reliable nature of Infotility, based on the fact that the content was reviewed by specialists and because their clinics were partners in the project. This was one of the most appreciated characteristics of Infotility. This corroborates previous research revealing that credibility was the most important attribute for informational websites (Kahlor & Mackert, 2009). This suggests that the involvement of physicians and clinics in the development of information material should be promoted.

The most common theme of our qualitative analysis was that participants would have liked more detailed information, particularly about the medical aspects of infertility. In previous literature, being more educated, being younger, and being a woman have been associated with greater information needs (Suhonen et al., 2005). Our quantitative results do not confirm trends with regard to age and sex but do confirm a link between information needs and educational level. However, it is interesting to note that there were participants with unmet information needs in all categories of education, and that the higher education group (graduate studies) had a smaller proportion of participants with unmet needs than the undergraduate studies group. This shows that despite the existence of a trend, education should not be used as a direct proxy for estimating information needs.

Ethnicity was linked to having unmet information needs with white participants being more likely to have unmet needs after using Infotility than non-white participants, which is in line with previous research (Kahlor & Mackert, 2009). This could be because non-white patients are often given less health information by healthcare providers (Shen et al., 2018), or perhaps white patients were more likely to have sought medical information online already. Research is required to understand such ethnicity-based differences in doctor-patient relationships as well as in information needs in general. Nonetheless, the existence of ethnicity-based differences in our study reinforces the need for flexible tools tailored to user preferences.

Participants in our qualitative survey conveyed the idea that the amount of information provided on Infotility was sufficient for patients at the beginning of their fertility journey, but insufficient for those who had been in treatment for long periods and already had the time to acquire information. This echoes the idea that the internet has allowed patients to become ‘experts’ on their own conditions and treatments (Dedding, van Doorn, Winkler, & Reis, 2011). It has been suggested that this ‘expert’ status creates a need for more complex and elaborate information tools (Coulter, 1998). However, when we compared the average amount of time that had passed since our participants had first visited a fertility specialist, we found no significant difference between the two groups. This suggests that patients with high information needs can become ‘experts’ on their condition and treatment very soon after they start their fertility journey. Therefore, time in treatment is not necessarily a good predictor of information needs. This reinforces the importance of individual preferences and coping mechanisms in the definition of information needs as highlighted by Timmins (2006).

These findings suggest that information needs are more importantly defined by factors other than demographics. The models proposed by Miller (1995) and Timmins (2006) both support the idea that the provision of information should be tailored to the coping mechanisms of the patient, with ‘monitors’ and ‘problem-focused’ patients generally requiring more extensive information than ‘blunters’ and ‘emotion-focused’ patients. It has been shown that monitors have decreased stress levels when they are given a large amount of information about procedures (Miller, 1995).

In our group with unmet information needs, many formulated their dissatisfaction with the amount of information on the app by saying that they ‘had seen it all’ in very little time. In addition, this group gave the app lower ratings with regard to quantity of information, helpfulness of individual pages, and overall star-rating. Surprisingly, our engagement analysis showed that this group actually viewed more pages and spent more time on each page than the met needs group. This suggests that they are very motivated and invested in their search for detailed information, and that they had high expectations with regard to the information that Infotility could provide. It is possible that our unmet needs group comprised more ‘monitors’ and ‘problem-focused’ copers that our met needs group did.

Participants mentioned that the information they received from fertility specialists was often insufficient and that this may have prevented them from making an informed choice. Our participants also said that Infotility provided them with enough knowledge to carry more informed discussions with their physician and ask for alternative treatments. This is consistent with the results of Kahlor and Mackert (2009) who found that online information-seeking was correlated with a sense of being informed, a sense of having made better decisions, and more appreciation of doctor-patient communication. Slauson-Blevins, McQuillan, and Greil (2013) found that people seeking fertility services use the internet as a complement to the information already provided by physicians, not as a substitute. This should reassure practitioners who hesitate to encourage patients to find information online. Clinics could provide a list of reliable websites or mobile applications that patients can use as complements to the information they receive in clinics.

Participants from the unmet needs group had mentioned dissatisfaction with the information provided at the clinic as a motivation for seeking information online. Therefore, we were expecting that this group might score lower than the met needs group in terms of satisfaction with the information provided at the clinic. However, these scores did not significantly differ. This might be attributable to the fact that conversations with physicians are more adaptable to each patient’s level of knowledge and understanding. A previous study has shown that fertility patients who feel they can rely on the physician for information rely less on the internet for information (Kahlor & Mackert, 2009). This highlights the importance of making internet tools adaptable as well, as our participants also mentioned that health care providers do not always have the time to have these long conversations.

### Implications for readability strategies in health communication

Our study shows that fulfilling information needs is important, not only to achieve ideals of patient-centered care and informed consent, but also to contribute to psychological well-being. Health care agencies generally agree that health information tools should aim for a Flesh Kincaid Grade Level of no more than Grade 6–8 to ensure that they can be understood by a large majority of users. However, our results tend to show that this one-size-fits-all approach does not meet the information needs of the most engaged or curious users. Infotility was designed at a reading level slightly above the most common recommendations. As previously mentioned, the number of syllables per word is the basis for computing reading levels. A more difficult reading level allows for more medical terms to be used, because medical terms often have several syllables. Despite this, many participants believed that there was not enough detail, especially medical, for the app to meet their needs. Research has shown that people with education below grade 8 will retain more information from material designed at a grade 6 level than at grade 9 or 12. However people with higher education (grade 9 and above) learn more from material designed at grade 9 level and even more from material designed at grade 12 level (Dowe, Lawrence, Carlson, & Keyserling, 1997). Similarly there is a positive relationship between education level and subjective perception of benefit derived from material at grade 12 reading level (French & Larrabee, 1999).

In the context of fibromyalgia, it has been shown that higher quality information websites were written at a more difficult reading level (grade 11) and lower quality websites were written at an easier reading level (grade 6-8), with exhaustiveness and readability of the information among the criteria for quality determination (Daraz et al., 2011). It is not clear whether simplification of the discourse necessarily implies a lack of detailed information, and more specifically, subjective satisfaction of readers with regard to detail. However, some literature from the field of scientific popularization seem to indicate that it may be the case. In addition to the above-mentioned problem with the number of syllables in medical terms, there is also a length consideration in endeavors to simplify very specific technical information. A specific scientific term usually cannot be replaced by a single simpler term. Defining such complex terms demand using a number of approximately similar simpler terms and explaining how they relate (Mortureux, 1984). In addition, explaining a scientific phenomenon in lay terms demands using a series of definitions, comparisons, analogies and metaphors (Landry, 1992). This requires a considerable amount of text. Therefore, a lay-term version is likely to be longer than the more scientific version of the same content. This is problematic because informational material is also required to be limited in the length of paragraphs and sections (JGH Patient Education Network Working Group, 2008). A similar debate in the field of law suggests that simplifying some complex issues may result in a loss of accuracy or meaning (Assy, 2011; Masson & Waldron, 1994). Therefore, it might be difficult to satisfy the information needs of some patients without departing from common literacy level guidelines.

Our results also show that demographic factors, including education, cannot be taken as direct proxies to estimate information needs. Therefore, what is needed might not be a change in the recommended readability levels. Instead, adaptability could be introduced in information tools. French and Larrabee (1999) suggested preparing different materials (grade 4, 8 and 12) and offering them according to patients’ needs. eHealth today provides the opportunity to develop more flexible tools that can be responsive to patients’ desired level of detail by providing optional ‘learn more’ bubbles with more specific information as well as links to external websites. Accordingly, the next version of Infotility is being designed with optional sections that, when opened, offer more detail and higher complexity levels on the topic. This ensures that people with low reading levels understand the basic information provided without feeling overwhelmed, while more health literate or curious users may also have their needs satisfied.

### Strengths and limitations

Participants were recruited from fertility clinic waiting rooms; therefore, results cannot generalize to those who do not seek fertility treatment. This limits our sample, as we likely excluded those who may not be able to afford fertility treatment. Infertile couples who do not seek treatment are more likely to be non-white and less educated and to have a lower income (Greil & McQuillan, 2004; Greil, Johnson, Lowry, McQuillan, & Slauson-Blevins, 2020). In addition, this study only considers the information needs of those individuals who finished the study and left qualitative responses to follow-up questions. This excludes participants who dropped out and did not complete follow-up questionnaires after using the app. It is possible that those who were most dissatisfied with the app did not finish the study. Although coding was based on explicit written information, it is possible that some participants hold different views about their need satisfaction than was coded for in the present study.

Despite the limitations, the present study has important strengths. Our sample was diverse which improves generalizability to Canadian people seeking fertility services. Mixed methods are considered to generate a more comprehensive understanding of a given issue compared to performing qualitative or quantitative research alone (Creswell, 2014). Finally, the need to involve patient representatives in the development of informational material has been stressed many times in the literature (Coulter, 1998; Daraz et al., 2011). This study takes part in this trend, by highlighting the importance of using feedback from patients when developing online health information. The qualitative results of this study are being considered for fine-tuning the next version of Infotility.

## Conclusion

People seeking fertility services may vary in the amount of diagnostic and treatment information they wish to receive. Ethnicity and education level might be an indicator of greater information needs but should not be used as a proxy in and of themselves. In our study, meeting information needs was associated with improved fertility-related quality of life (emotional subscale), reduced anxiety and improved sense of empowerment. Adaptable tools that fulfill the needs of the most knowledgeable and curious patients without de facto overwhelming the patient with lesser literacy and interest may contribute to improved sense of empowerment and autonomy and contribute to coping with fertility treatments. While this is particularly relevant to highly literate patient populations such as people seeking fertility services, it is also applicable to other patient populations. Further research on the relationships between health literacy and the fulfillment of information needs could inform the development of such adaptable tools.

## Note

1 For the purposes of this paper, ‘active’ app users are all those who visited *at least one page* on the app not including the Dashboard.
